# Drought Stress Results in a Compartment-Specific Restructuring of the Rice Root-Associated Microbiomes

**DOI:** 10.1128/mBio.00764-17

**Published:** 2017-07-18

**Authors:** Christian Santos-Medellín, Joseph Edwards, Zachary Liechty, Bao Nguyen, Venkatesan Sundaresan

**Affiliations:** a Department of Plant Biology, University of California, Davis, California, USA; b Department of Plant Sciences, University of California, Davis, California, USA; Massachusetts General Hospital

**Keywords:** drought, endosphere, microbiome, rhizosphere, rice, roots

## Abstract

Plant roots support complex microbial communities that can influence plant growth, nutrition, and health. While extensive characterizations of the composition and spatial compartmentalization of these communities have been performed in different plant species, there is relatively little known about the impact of abiotic stresses on the root microbiota. Here, we have used rice as a model to explore the responses of root microbiomes to drought stress. Using four distinct genotypes, grown in soils from three different fields, we tracked the drought-induced changes in microbial composition in the rhizosphere (the soil immediately surrounding the root), the endosphere (the root interior), and unplanted soils. Drought significantly altered the overall bacterial and fungal compositions of all three communities, with the endosphere and rhizosphere compartments showing the greatest divergence from well-watered controls. The overall response of the bacterial microbiota to drought stress was taxonomically consistent across soils and cultivars and was primarily driven by an enrichment of multiple *Actinobacteria* and *Chloroflexi*, as well as a depletion of several *Acidobacteria* and *Deltaproteobacteria*. While there was some overlap in the changes observed in the rhizosphere and endosphere communities, several drought-responsive taxa were compartment specific, a pattern likely arising from preexisting compositional differences, as well as plant-mediated processes affecting individual compartments. These results reveal that drought stress, in addition to its well-characterized effects on plant physiology, also results in restructuring of root microbial communities and suggest the possibility that constituents of the altered plant microbiota might contribute to plant survival under extreme environmental conditions.

## IMPORTANCE

With the likelihood that changes in global climate will adversely affect crop yields, the potential role of microbial communities in enhancing plant performance makes it important to elucidate the responses of plant microbiomes to environmental variation. By detailed characterization of the effect of drought stress on the root-associated microbiota of the crop plant rice, we show that the rhizosphere and endosphere communities undergo major compositional changes that involve shifts in the relative abundances of a taxonomically diverse set of bacteria in response to drought. These drought-responsive microbes, in particular those enriched under water deficit conditions, could potentially benefit the plant as they could contribute to tolerance to drought and other abiotic stresses, as well as provide protection from opportunistic infection by pathogenic microbes. The identification and future isolation of microbes that promote plant tolerance to drought could potentially be used to mitigate crop losses arising from adverse shifts in climate.

## INTRODUCTION

Plants are intimately linked to the microbial communities that inhabit the soil-root continuum: not only do roots shape the environment where these communities establish, but the associated microorganisms can influence the nutrition, health, and overall fitness of their plant hosts (1). Extensive characterizations of root-associated microbiomes across various plant systems have yielded valuable insights into the factors affecting community assembly (2–7). Microbial diversity has been shown to follow a compositional transition from the outside to the inside of the root, with four distinct compartments identified along this gradient: bulk soil (not affected by root activity), rhizosphere (the soil microenvironment immediately surrounding the root), rhizoplane (the root surface), and endosphere (the root interior) (8). The specific compositions of these compartments depend upon soil source as root-associated microorganisms are predominantly acquired from the surrounding edaphic communities. Additionally, plant genotype is responsible for some of the variation observed in root microbiomes, suggesting an active role of the host in the establishment of the communities.

With these advances in our understanding of root microbiome structure, it is of interest to dissect the effect of extreme environments on community composition to help reveal how the network of plant-microbiome interactions is reshaped under challenging conditions (9). Furthermore, identification of root-associated microorganisms that thrive under adverse environments could lead to the discovery of beneficial symbioses since microbial traits that confer stress tolerance could potentially be advantageous to the host (10). Drought represents one of the major threats to food security as it can drastically decrease plant yield and lead to land degradation (11). Moreover, the frequency of droughts is projected to increase by the end of this century (12), a trend that could progressively alter the subterranean properties of affected agroecosystems. While drought has been shown to restructure the bacterial diversity in soils (13, 14), little is known about its influence on the communities assembled in the rhizocompartments of plants. These communities could be directly affected by drought stress and also by host-mediated processes as water deficit triggers a cascade of molecular, physiological, and developmental responses in plants (15, 16). Roots, in particular, can change their architecture (17) and resource allocation (18) to avoid dehydration. Root exudation profiles can also shift under stress (19, 20), potentially affecting rhizospheric properties.

In this study, we have examined the impact of drought on the root-associated microbiomes of cultivated rice. Rice is not only one of the major staple foods, but given its semiaquatic growth habitat, it is highly susceptible to water deficit. In particular, we have explored the compositional shifts in the rhizosphere, endosphere, and bulk soil communities of a diverse set of rice accessions grown in different soils. This approach has allowed us to assess the extent and conservation of the drought-mediated changes experienced by the root microbiota.

## RESULTS

Root-associated microbiomes are spatially structured in distinct compartments whose compositions are affected by several factors, including soil type and plant genotype. Therefore, to examine the microbial community response to drought stress, we designed a multifactorial experiment to reveal interactions between changes in microbiota composition, if any, with these key determinants of root microbiome assembly (see Fig. S1A and B in the supplemental material). Briefly, we grew four cultivated rice varieties in three distinct soils under controlled greenhouse conditions. The set of chosen cultivars broadly covered the phylogenetic spectrum of domesticated rice with two *Oryza glaberrima* accessions: TOg7102 (G1) and CG14 (G2), and two *Oryza sativa* varieties: *indica* IR20 (S1) and *japonica* M206 (S2). Among these cultivars, CG14 has been previously identified as drought resistant (21). For this experiment, soils were collected from rice fields at three separate locations across the California Central Valley (Arbuckle, Davis, and Biggs [Fig. S1C]) in order to obtain a variant set of edaphic communities encountered by cultivated rice in this region (5). Drought was imposed on 1-month-old plants by ceasing irrigation and letting the soils progressively dry down until half of the plants exhibited leaf rolling, a common symptom of drought stress (22). At this point, sufficient water was added periodically to keep the plants alive but still under stress (see Fig. S2 in the supplemental material). Three weeks after water withdrawal, soil from treated samples exhibited an average reduction of 61% in moisture content relative to control samples that were kept under well-irrigated conditions (see Table S1 in the supplemental material). The amount of water lost varied between soil types and cultivars, with the *O. sativa* varieties retaining the highest percentage of moisture by the end of the experiment. In contrast, bulk soils that underwent the same dry-down regime lost an average of 9% moisture during that period, showing that plants drastically increased the desiccation rate in planted soils. It is important to note that the drought-stressed plants remained viable throughout the experiment, as demonstrated by their rapid response to watering with loss of the visible drought symptoms.

10.1128/mBio.00764-17.2 FIG S1  Experimental design. (A, B, and D) Watering treatment was assigned to main plots (A), and combinations of cultivar and soil types were assigned to subplots (B). (C) Locations of rice fields where soils were collected. (E) Section of the root sampled for DNA extraction. Download FIG S1, EPS file, 0.4 MB. Copyright © 2017 Santos-Medellín et al. 2017 Santos-Medellín et al. This content is distributed under the terms of the Creative Commons Attribution 4.0 International license.

10.1128/mBio.00764-17.3 FIG S2  Representative set of cultivars TOg7102 (A), CG14 (B), IR20 (C), and M206 (D) at the end of the experiment. Download FIG S2, PDF file, 0.5 MB. Copyright © 2017 Santos-Medellín et al. 2017 Santos-Medellín et al. This content is distributed under the terms of the Creative Commons Attribution 4.0 International license.

10.1128/mBio.00764-17.9 TABLE S1  (A) Soil water content of drought and control samples at the end of the experiment. (B) Analysis of variance (ANOVA) testing the effect of watering treatment, soil type, and cultivar on the water content of soils at the end of the experiment. Bulk soil samples were excluded before running the analysis. Download TABLE S1, PDF file, 0.1 MB. Copyright © 2017 Santos-Medellín et al. 2017 Santos-Medellín et al. This content is distributed under the terms of the Creative Commons Attribution 4.0 International license.

In order to examine the root-associated microbiome response to water deficit, we sampled roots immediately below the root-shoot junction (Fig. S1E). This allowed us to assess the compositional transitions of communities already assembled in roots rather than changes in the colonization of new root tissue. We profiled the communities assembled in the rhizosphere and endosphere, as well as in unplanted bulk soils, via high-throughput sequencing. In particular we amplified the V4 region of the 16S rRNA gene to survey the bacterial and archaeal diversity. After removing chimeric and organellar sequences and filtering out operational taxonomic units (OTUs) not present in at least 5% of our samples, we identified 12,892 OTUs (mean of 29,910 reads per sample).

### Root bacterial and archaeal communities exhibit drought-mediated compositional shifts.

**FIG 1:**
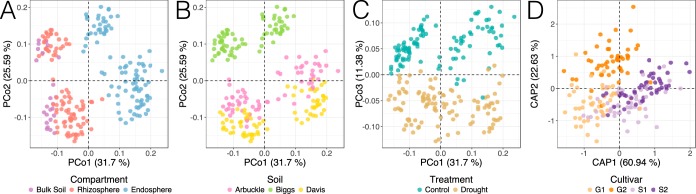
Compartment, soil type, drought treatment, and cultivar shape the overall composition of root-associated bacterial and archaeal communities. (A to C) Unconstrained analysis of principal coordinates. (D) Partial canonical analysis of principal coordinates constrained to cultivar effect while controlling for soil, treatment, and compartment effects. Orange points correspond to *O. glaberrima* varieties (G1, TOg7102; G2, CG14), while purple ones correspond to *O. sativa* varieties (S1, IR20; S2, M206). All analyses were performed on weighted UniFrac distances.

We analyzed the impact of each of the experimental factors and their interactions on the overall composition by performing a permutational multivariate analysis of variance (PERMANOVA) on weighted UniFrac distances (see Table S2 in the supplemental material). The results showed that all of the main experimental factors significantly impacted the rice root-associated bacterial and archaeal communities, with compartment representing the main source of variation (*R*^2^ = 0.285, *P* < 0.001), followed by soil type (*R*^2^ = 0.277, *P* < 0.001), drought treatment (*R*^2^ = 0.099, *P* < 0.001), and cultivar (*R*^2^ = 0.023, *P* < 0.001). An unconstrained principal-coordinate analysis (PCoA) confirmed some of these findings (Fig. 1A to C): while the first principal coordinate (PCo1) displayed the compositional transition across the soil-root continuum (spatial compartmentalization), the second one separated the communities by soil type. Moreover, the third axis exposed a clear distinction between drought-treated versus control samples. Cultivar differences were not immediately evident in the unconstrained ordination but were uncovered by a canonical analysis of principal coordinates (CAP) that partialed out the other experimental variables (Fig. 1D). This approach revealed a separation between *O. sativa* and *O. glaberrima* cultivars, a result that mirrors patterns previously reported for rice (5).

10.1128/mBio.00764-17.10 TABLE S2  (A) PERMANOVA testing the effect of soil, compartment, and watering treatment on beta-diversity. (B) Simple effects testing the effect of drought within each level of compartment. (C) PERMANOVA testing the effect of soil, compartment, watering treatment, and cultivar on beta-diversity. Because bulk soils do not have a cultivar assigned, they were excluded before running the analysis. All analyses were performed on weighted UniFrac distances. Download TABLE S2, PDF file, 0.03 MB. Copyright © 2017 Santos-Medellín et al. 2017 Santos-Medellín et al. This content is distributed under the terms of the Creative Commons Attribution 4.0 International license.

The PERMANOVA also detected a significant interaction between compartment and treatment (*R*^2^ = 0.020, *P* < 0.001), suggesting a potential differential response to water deprivation across compartments. To further interrogate this possibility, we performed a partial CAP that assessed the variation due to compartment, drought treatment, and their interaction, while removing the effect of soil type (see Fig. S3 in the supplemental material). The constrained ordination showed that the divergence between treated and control communities was much higher in the rhizocompartments than in the bulk soil communities. This observation was further supported by a simple effect analysis that split the effect of drought treatment by each compartment (Table S2): while drought significantly affected the composition of the communities assembled in all compartments, the effects were stronger in the endosphere (*R*^2^ = 0.059, *P* = 0.003) and rhizosphere (*R*^2^ = 0.052, *P* = 0.003) than in the bulk soil (*R*^2^ = 0.008, *P* = 0.003). Given that bulk soil samples retained much higher levels of moisture than planted soils (Table S1), it is likely that the relatively strong rhizospheric community response is, in part, a consequence of the differences in water deficit experienced by that community. Plant processes could also be contributing to and amplifying the changes in the rhizospheric and endospheric communities.

10.1128/mBio.00764-17.4 FIG S3  Partial canonical analyses of principal coordinates constrained to compartment and treatment while controlling for soil effect. CAP was performed on weighted UniFrac distances. Download FIG S3, EPS file, 0.6 MB. Copyright © 2017 Santos-Medellín et al. 2017 Santos-Medellín et al. This content is distributed under the terms of the Creative Commons Attribution 4.0 International license.

### Differential abundance of bacterial taxa under drought.

In order to identify taxa that were enriched or depleted in drought-stressed communities, we fitted negative binomial models to the abundances of individual taxa and evaluated differential abundance patterns between treated and control samples using the Wald test. These analyses were performed at the phylum and OTU levels to assess the extent of these changes at different taxonomic ranks.

### Phylum-level analysis.

**FIG 2:**
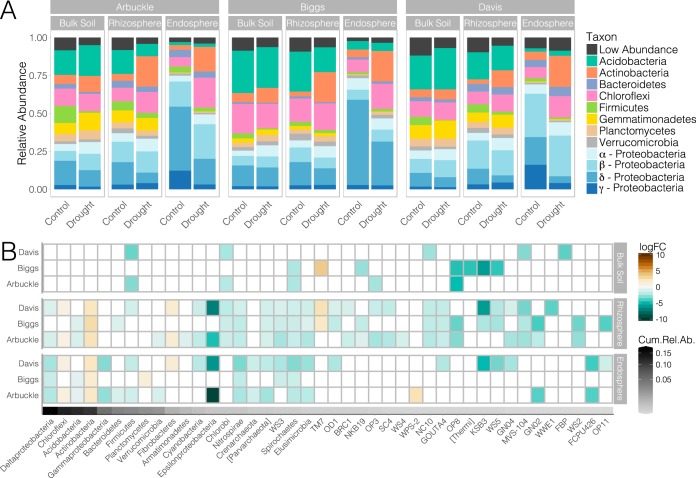
Drought affects the relative abundances of bacterial and archaeal higher taxa. (A) Relative abundances of the most abundant phyla and *Proteobacteria* classes in each compartment, soil, and drought treatment. (B) Drought-responsive taxa (*P* < 0.05) in each compartment and soil type. The color of the cell indicates the log_2_ fold change in relative abundance with respect to the control treatment: an increase tends toward brown, while a decrease tends toward green. Taxa are ranked by the cumulative relative abundance in the whole data set as indicated by the gray scale at the bottom of the plot. The plot displays all taxa detected as significantly affected by drought treatment in at least one compartment or soil type.

First, we evaluated the main effects of drought treatment and the other experimental variables on the abundances of bacterial and archaeal phyla. Since 45% of the reads in our 16S data set were identified as *Proteobacteria*, we split this group into its respective classes (Fig. 2A). Out of 59 higher taxa, 38 were significantly influenced by water deprivation (see Fig. S4A in the supplemental material). Moreover, the cumulative relative abundance (CRA) of this set of microbes amounted to 70% (Fig. S4B). In comparison, spatial compartmentalization, soil type, and cultivar influenced 44 (95% CRA), 43 (80% CRA), and 10 (5% CRA) taxa, respectively. Thus, while compartment and soil source remained the primary determinants, drought was also a major factor affecting the relative abundances of higher taxa. We further inspected the drought-mediated changes within individual compartments in each of the soils (Fig. 2B). Regardless of soil type, drought affected only a few low-abundance phyla in the bulk soils, whereas it shifted major and minor groups in the rhizospheric and endospheric compartments. Out of all the phyla affected, 6 were significantly enriched under drought stress and 35 were significantly diminished, indicating a broad exclusionary effect of drought at this taxonomic level. Furthermore, for phyla detected as significant across multiple tests, the direction of the response (increase or decrease in relative abundance after the drought treatment) was always consistent. Among this group, various highly abundant taxa were affected in all or most rhizocompartments and soils, including *Actinobacteria* and *Chloroflexi* (both significantly enriched under drought), as well as *Deltaproteobacteria* and *Acidobacteria* (both significantly depleted under drought). Additionally, several low-abundance taxa (*Nitrospirae*, *Parvarchaeota*, WS3, *Spirochaetes*, and *Elusomicrobia*) were also consistently depleted under drought. Thus, the core response to drought at the phylum level comprised a diverse set of taxa that included some of the most prominent members of rice root communities.

10.1128/mBio.00764-17.5 FIG S4  Compartment, soil, drought treatment, and cultivar affect the relative abundances of higher taxa. (A) Main effects on the relative abundances of higher taxa in bacterial and archaeal communities. The color in the cell displays the −log_10_ adjusted *P* value of the differentially abundant (*P* < 0.05) taxa. (B) Cumulative relative abundance of the taxa affected by each factor. Download FIG S4, EPS file, 0.5 MB. Copyright © 2017 Santos-Medellín et al. 2017 Santos-Medellín et al. This content is distributed under the terms of the Creative Commons Attribution 4.0 International license.

### OTU-level analysis.

**FIG 3:**
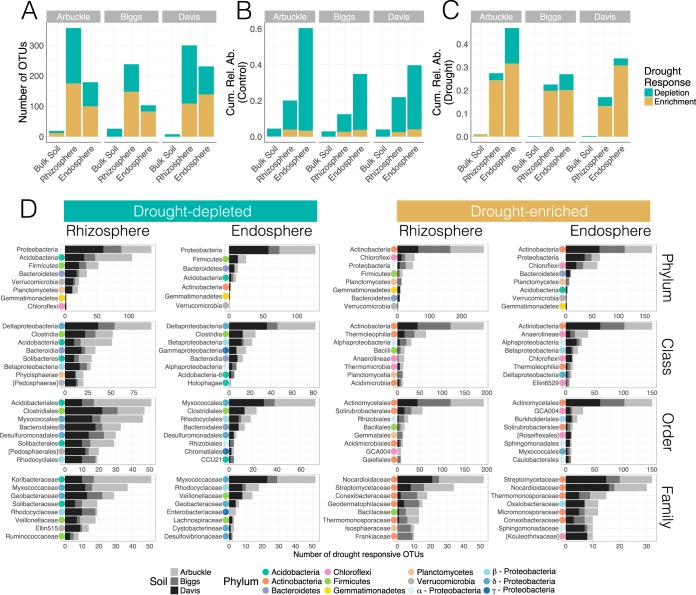
Drought affects a taxonomically diverse array of OTUs. (A) Number of OTUs detected as differentially abundant (*P* < 0.05) between control and drought-treated samples. (B and C) Cumulative relative abundances of drought-responsive OTUs in well-watered (B) and drought-stressed (C) communities. (D) Phyla, classes, orders, and families most highly represented in the set of drought-responsive OTUs. The colored point to the left of each bar indicates the phylum or *Proteobacteria* class to which the taxon belongs.

To identify the OTUs driving these taxonomic shifts, we assessed the impact of water deprivation on the abundances of individual OTUs. Similar to the analysis carried out at the phylum level, we performed tests for each compartment within each soil type. Across all these contrasts, we detected 1,461 drought-responsive OTUs that spanned 31 phyla, 62 classes, 87 orders, and 111 families (Fig. 3; see Data Set S1 in the supplemental material). Out of these differentially abundant OTUs, 52% were enriched under drought, while 48% were depleted. Between compartments, the rhizosphere communities presented the highest number of differentially abundant OTUs, followed by the endosphere and bulk soil communities (Fig. 3A). Nevertheless, the drought-responsive OTUs detected in the endospheric compartment accounted for the highest cumulative relative abundances within their respective communities, a pattern that was consistent across all soil types (Fig. 3B and C).

10.1128/mBio.00764-17.1 DATA SET S1  List of OTUs detected as differentially abundant between control and drought samples within each soil-compartment combination. The estimate column represents the log_2_ fold change in relative abundance with respect to water controls. Adjusted *P* values were calculated using the Bonferroni correction for multiple comparisons. Download DATA SET S1, TXT file, 0.3 MB. Copyright © 2017 Santos-Medellín et al. 2017 Santos-Medellín et al. This content is distributed under the terms of the Creative Commons Attribution 4.0 International license.

Among drought-enriched OTUs, *Actinobacteria* was, by far, the most highly represented phylum in both rhizosphere and endosphere communities (Fig. 3D). In particular, multiple families of the order *Actinomycetales* were identified across all soil types. Various *Actinobacteria* OTUs belonging to the *Thermoleophilia* and *Acidimicrobiia* classes were also enriched, although most of them were detected in the rhizosphere. Thus, the ubiquitous *Actinobacteria* increase observed at the phylum level stemmed from the simultaneous enrichment of a taxonomically diverse set of OTUs. Additionally, the relative abundances of several OTUs classified as *Chloroflexi*, mainly from the class *Anaerolineae*, increased under water deficit. Within *Proteobacteria*, most drought-enriched OTUs were classified as *Alphaproteobacteria* and *Betaproteobacteria*. *Alphaprotebacteria* was mainly represented by the orders *Sphingomonadales*, *Caulobacterales* (both mostly in the endosphere), and *Rhizobiales* (mostly in the rhizosphere), while *Betaproteobacteria* was mostly represented by the order *Burkholderiales*. Finally, in the rhizosphere, drought led to an enrichment of OTUs belonging to classes *Bacilli* (phylum *Firmicutes*) and *Planctomycetia* (phylum *Planctomycetes*).

The phylum with the most drought-depleted OTUs across both rhizocompartments was *Proteobacteria* (Fig. 3D). The majority of these OTUs were classified as *Deltaproteobacteria*, with *Myxococcales* and *Desulfuromonadales* being the orders most broadly affected. OTUs from the order *Rhodocyclales* (class *Betaproteobacteria*) were also consistently depleted by drought in all soils and rhizocompartments. In the rhizosphere, *Acidobacteria* was the second most highly represented phylum within the drought-depleted set. Specifically, various OTUs belonging to classes *Acidobacteria* and *Solibacteres* were detected. Lastly, multiple OTUs identified as *Clostridia* (phylum *Firmicutes*), *Bacteroidia* (phylum *Bacteroidetes*), and *Phycisphaerae* (phylum *Planctomycetes*) were also depleted under drought: the first two groups were detected in both compartments, while the last one was detected mainly in the rhizosphere.

### Coherence of drought response across taxonomic ranks.

**FIG 4:**
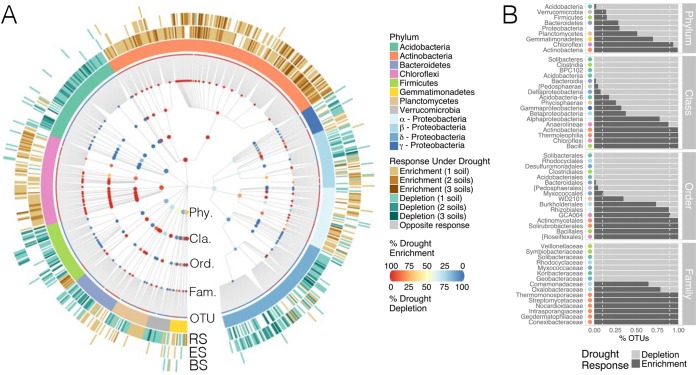
Drought response is generally coherent within higher taxa. (A) Taxonomy dendrogram displaying the drought-responsive OTUs (*P* < 0.05) detected across all compartments and soil types. Only the OTUs belonging to the most represented higher taxa (innermost circle) are shown, while a complete list of all the differentially abundant OTUs is provided in Data Set S1. The three outermost rings indicate the number of soil types in which the relative abundance of an OTU was significantly higher (brown) or lower (green) under drought in the rhizosphere (RS), endosphere (ES), and bulk soil (BS) communities. The nodes in the cladogram indicate the phylum (Phy.), class (Cla.), order (Ord.), and family (Fam.) to which each OTU belongs. The color of the node represents the response coherence (measured as the percentage of OTUs enriched or depleted under drought) within the subtree rooted at that node: consistent enrichment under drought tends toward red, while consistent depletion tends toward blue. (B) Percentage of OTUs within individual taxa that were enriched or depleted under drought stress. Only taxa with more than 15 OTUs are shown. The colored point to the left of each bar indicates the phylum or *Proteobacteria* class to which the taxon belongs.

Comparison of the results obtained at the phylum- and OTU-level analyses (Fig. 2 and 3) shows that some of the major taxonomic trends were consistent in both cases: *Actinobacteria* and *Chloroflexi* were enriched under drought, while *Deltaproteobacteria* and *Acidobacteria* were depleted. On the other hand, some taxa not detected as responsive at the phylum level were nevertheless highly represented in the set of drought-responsive OTUs (e.g., *Alphaproteobacteria*). Furthermore, while the overall relative abundances of certain phyla were significantly lower under drought, several OTUs within those phyla responded in the opposite direction (e.g., within the *Firmicutes*). This last observation highlights the importance of assessing the consensus in the response to drought stress as it can reveal the ecological and functional coherence of higher taxonomies (23). To address this question, we calculated the percentage of drought-responsive OTUs whose relative abundances shifted in the same direction (i.e., enrichment or depletion under drought) within major taxa (Fig. 4). At the phylum level, *Actinobacteria* and *Chloroflexi* showed the greatest consensus toward drought enrichment as more than 90% of their OTUs followed that trend. In contrast, *Acidobacteria* was the only phylum with more than 90% OTUs decreasing under drought. At lower taxonomic ranks, various classes and orders were highly coherent despite belonging to phyla with low consensus in their response to drought. For example, within *Proteobacteria*, class *Deltaproteobacteria* and order *Rhodocyclales* (class *Betaproteobacteria*) were persistently depleted under drought. Among *Firmicutes*, class *Clostridia* diminished under drought, whereas class *Bacilli* increased. Within *Planctomycetes*, all *Planctomycetia* were enriched, and among *Bacteroidetes*, most *Bacteroidia* were depleted. Together, these results suggest a strong taxonomic clustering in the responses of root-associated OTUs to drought.

### Interactions between drought treatment and rhizocompartment.

**FIG 5:**
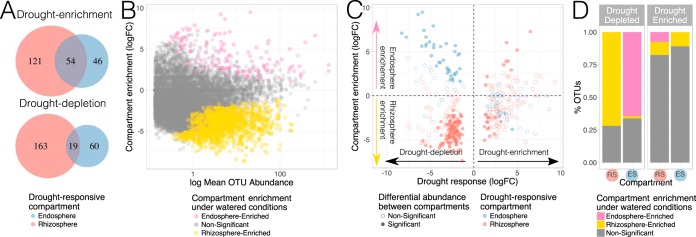
Rhizosphere and endosphere communities exhibit a distinct drought response. This figure only shows the results obtained for plants grown in Arbuckle soil. The rest of the soils displayed similar patterns (shown in Fig. S5). (A) Overlap of drought-responsive OTUs between compartments. (B) Mean OTU abundance plot displaying the patterns of abundance between rhizospheric and endospheric compartments in well-watered communities. The *x* axis displays the mean abundance of the OTUs, and the *y* axis displays the log_2_ fold change in relative abundance with respect to the rhizosphere: a positive value indicates enrichment in the endosphere, while a negative value indicates enrichment in the rhizosphere. (C) Relationship between drought response and differential abundance between compartments of drought-responsive OTUs. The *x* axis displays the log_2_ fold change in relative abundance with respect to the control treatment: a positive value represents drought enrichment, and a negative value represents drought depletion. The *y* axis displays the differential abundance patterns between compartments under well-watered conditions. (D) Proportion of drought-responsive OTUs that were differentially abundant between compartments under well-watered conditions.

We analyzed the interaction between drought and root compartments by exploring the overlap between the drought-responsive OTUs detected in the rhizosphere and endosphere. While there was a considerable overlap between the communities, the majority of OTUs affected by drought were usually compartment specific (Fig. 5A; see Fig. S5 in the supplemental material). A potential explanation for this differential response to drought is that certain OTUs were depleted or enriched in particular compartments before the communities were exposed to water deprivation. Under this scenario, differential abundance patterns between rhizospheric and endospheric communities, established during root microbiome assembly predetermined the set of OTUs that could have responded to drought in each compartment. We utilized the control samples to estimate the root assemblies prior to drought treatment, with the assumption that root communities of watered 4-week-old rice plants do not show much change during vegetative growth to 7-week-old plants (5). Specifically, we compared the rhizospheric and endospheric communities of well-watered samples to identify which OTUs significantly differed between these compartments. Overall, many more OTUs were enriched in the rhizosphere than in the endosphere regardless of soil source (Fig. 5B). This is consistent with previous findings showing that the acquisition of an endospheric microbiota involves a strong exclusion of soil microbes, probably occurring at the root surface (8). We then focused on the OTUs that were affected by water deprivation in only one compartment and assessed the relationship between their response to drought and compartment enrichment patterns (Fig. 5C and D). Among OTUs whose relative abundances decreased under drought, we found that most (70 to 75% [Fig. 5D]) were differentially abundant between compartments in well-watered communities. Specifically, the compartment in which they were enriched was correlated with the compartment in which they were exclusively depleted by drought: i.e., OTUs that were only drought depleted in the endosphere were, under well-watered conditions, predominantly enriched in that compartment relative to the rhizosphere. Thus, compartment-specific drought depletion likely resulted from differential abundances between compartments preceding the drought treatment. On the other hand, among the drought-enriched set, there was a much higher proportion (80 to 85% [Fig. 5D]) of OTUs whose relative abundances were not significantly different between rhizosphere and endosphere communities under well-watered conditions, a pattern observed across all three soils. Thus, despite being present in both compartments at similar abundances under well-watered conditions, these OTUs were differentially affected by drought based on their location. Among this group of OTUs, certain taxa were highly represented based on the specific compartment in which they were drought enriched (Fig. S5E). For example, most OTUs classified as orders GCA004 (*Chloroflexi*) and *Burkholderiales* (*Betaproteobacteria*) were detected in the endosphere, whereas most OTUs belonging to classes *Bacilli* (*Firmicutes*), *Planctomycetia* (*Planctomycetes*), *Thermoleophilia* (*Actinobacteria*), and *Acidimicrobiia* (*Actinobacteria*) were identified in the rhizosphere (Fig. S5E).

10.1128/mBio.00764-17.6 FIG S5  Rhizosphere and endosphere communities exhibit a distinct drought response in Biggs and Davis soils. (A) Overlap of drought-responsive OTUs between compartments. (B) Mean OTU abundance plot displaying the patterns of abundance between rhizospheric and endospheric compartments in well-watered communities. The *x* axis displays the mean abundance of the OTUs, and the *y* axis displays the log_2_ fold change in relative abundance with respect to the rhizosphere: a positive value indicates enrichment in the endosphere, while a negative value indicates enrichment in the rhizosphere. (C) Relationship between drought response and differential abundance between compartments of drought-responsive OTUs. The *x* axis displays the log_2_ fold change in relative abundance with respect to the control treatment: a positive value represents drought enrichment, and a negative value represents drought depletion. The *y* axis displays the differential abundance patterns between compartments under well-watered conditions. (D) Proportion of drought-responsive OTUs that were differentially abundant between compartments under well-watered conditions. (E) Most highly represented orders in the set of OTUs detected as not differentially abundant between compartments but that were drought enriched in a compartment-specific manner. The left bar plots represent the percentage of OTUs that were drought enriched in each compartment, and the right bar plots show the number OTUs detected in each soil. Download FIG S5, EPS file, 2.7 MB. Copyright © 2017 Santos-Medellín et al. 2017 Santos-Medellín et al. This content is distributed under the terms of the Creative Commons Attribution 4.0 International license.

### Interactions between drought treatment and genotype.

Although plant genotypes had a relatively modest effect on the overall composition of root-associated microbiota (Table S2), they could have still influenced responses of individual OTUs to drought. To evaluate this possibility, we assessed the interactive effect of cultivar and watering regime on the relative abundances of OTUs. Across all these comparisons, we only detected 34 significant interactions (see Fig. S6 in the supplemental material)—all in the rhizosphere. This compartment-specific cultivar effect on drought response is consistent with previous results in rice showing that host genotype influences microbial composition mostly in the rhizosphere (5). Furthermore, these interactions were only observed in communities from plants grown in the Davis and Biggs soils, with no overlap between the OTUs detected in both soils. This result indicates that the interactive effect was highly dependent on the edaphic conditions experienced by the plant and root-associated microbiome. A closer inspection of the particular shifts in these OTUs revealed that, in most cases, OTUs experienced a stronger drought effect in the *O. glaberrima* cultivars than in the *O. sativa* cultivars. This contrasting effect between rice species could have resulted from differences in the microbiome assembled in each cultivar or from host-specific drought responses. Additionally, these cultivar-treatment interactions could also be related to differences in the percentage of soil moisture retained by each cultivar as *O. glaberrima* samples showed a stronger decrease in water content than *O. sativa* samples (Table S1). To summarize, the genotype-drought interactions are relatively minor, such that the overall effects of drought stress on microbiome composition are consistent for the different varieties of cultivated rice.

10.1128/mBio.00764-17.7 FIG S6  OTUs whose relative abundances were significantly affected by the interaction between drought and cultivar in the rhizosphere communities of plants grown in Biggs (A) and Davis (B) soils. The points on the *x* axis display the log_2_ fold change in relative abundance with respect to water controls. Only significant effects (*P* < 0.05) are shown. Download FIG S6, EPS file, 1.1 MB. Copyright © 2017 Santos-Medellín et al. 2017 Santos-Medellín et al. This content is distributed under the terms of the Creative Commons Attribution 4.0 International license.

### Root-associated fungal communities also exhibit shifts under drought stress.

We amplified and sequenced the internal transcribed spacer 1 (ITS1) to characterize the effect of drought on the fungal communities associated with rice roots. After discarding nonprevalent OTUs (see Materials and Methods), we identified 1,025 OTUs (mean of 38,034 reads per sample) clustered at a 97% similarity threshold. Even though the primer set we used was optimized for amplification of fungal ITS1 sequences (24, 25), OTUs without an assigned taxonomy represented a significant portion of these profiles: 435 OTUs that accounted for 20% of our data. Moreover, the distribution of unclassified OTUs varied widely across samples (see Fig. S7A and B in the supplemental material). Four out of the five most abundant unclassified OTUs returned fungal sequences as the top hits in BLAST searches (see Materials and Methods), consistent with their fungal origin. The lack of taxonomic information for a substantial portion of our data hindered the detailed characterization of drought-responsive fungi and their underlying phylogenetic patterns. Therefore, we restricted our analyses to an exploration of beta-diversity trends. We assessed the effect of the experimental variables in the overall composition of profiles that either included (INC) or excluded (EXC) the unclassified OTUs. PERMANOVA performed on Bray-Curtis dissimilarities revealed that, in both cases, the overall composition of the fungal communities was significantly affected by compartment (INC, *R*^2^ = 0.091, *P* < 0.001; EXC, *R*^2^ = 0.123, *P* < 0.001), soil type (INC, *R*^2^ = 0.329, *P* < 0.001; EXC, *R*^2^ = 0.332, *P* < 0.001), drought treatment (INC, *R*^2^ = 0.024, *P* < 0.001; EXC, *R*^2^ = 0.022, *P* < 0.001), and cultivar (INC, *R*^2^ = 0.013, *P* = 0.010; EXC, *R*^2^ = 0.013, *P* = 0.026). Unconstrained PCoA and partial CAP further confirmed these results (Fig. S7C to G), displaying similar patterns to the ones observed for the 16S profiles. Thus, despite the variation across compartments, soil types, and cultivars, the overall composition of the root-associated fungal communities is restructured by drought stress.

10.1128/mBio.00764-17.8 FIG S7  Analysis of fungal OTUs in watered and drought-treated samples. (A) Relative abundances of the most abundant higher taxa in each sample. The phyla *Ascomycota* (Asc.) and *Basidiomycota* (Bas.) have been split into their main classes. Color bars at the bottom indicate the cultivars from which the sample was collected: G1, TOg7102; G2, CG14; S1, IR20; and S2, M206. (B) Distribution of unassigned reads in the 16S and ITS1 data sets. (C to F) Compartment, soil type, drought treatment, and cultivar shape the overall composition of root-associated communities. (C to E) Unconstrained analyses of principal coordinates of ITS1 profiles with unclassified OTUs included (top set of panels) and excluded (bottom set of panels). (F) Partial canonical analyses of principal coordinates constrained to cultivar effect while controlling for soil, treatment, and compartment effects. (Top panels) Unclassified OTUs included. (Bottom panels) Unclassified OTUs excluded. All beta-diversity analyses were performed on Bray-Curtis dissimilarities. Download FIG S7, EPS file, 2.9 MB. Copyright © 2017 Santos-Medellín et al. 2017 Santos-Medellín et al. This content is distributed under the terms of the Creative Commons Attribution 4.0 International license.

## DISCUSSION

This study provides a detailed characterization of the effect of drought stress on root-associated microbiomes using the crop plant rice. Through a multifactorial greenhouse experiment that encompassed some of the main sources of variation in root communities, we were able to assess the extent and conservation of the microbial response across a representative compositional landscape. After a 3-week-long drought treatment, we observed a major shift in the bacterial, archaeal, and fungal communities assembled in the rhizospheric and endospheric compartments of four phylogenetically divergent rice varieties grown in three different soils. While poor taxonomic classification of fungal OTUs impeded a detailed investigation of taxa affected by drought stress, analysis of the bacterial communities revealed extensive taxonomic restructuring. This compositional transition involved significant changes in the relative abundances of a broad set of bacteria that spanned many prominent phyla and classes in the community. In particular, several OTUs belonging to the phyla *Actinobacteria* and *Chloroflexi*, as well as classes *Alphaproteobacteria*, *Bacilli*, and *Planctomycetia*, were significantly enriched under drought, whereas OTUs from the phylum *Acidobacteria* and classes *Deltaproteobacteria*, *Clostridia*, and *Bacteroidia* were generally depleted. These taxonomic trends were observed across all soil types despite their intrinsic compositional differences, indicating that drought stress had a reproducible effect on the root-associated communities of rice.

Assessment of the cohesiveness of drought response within higher taxa further revealed that shifts in relative abundances of these drought-responsive OTUs generally followed a consistent trend within certain phyla, classes, orders, and families. Such patterns could be reflecting the functional capabilities and life strategies shared by particular bacterial lineages (23, 26). For example, cell wall characteristics have previously been associated with desiccation tolerance. In particular, monoderm bacteria with thick cell walls have been shown to be better at resisting water stress (27). These properties might explain the conspicuous enrichment of *Actinobacteria* and *Chloroflexi* observed in drought-stressed communities. Interestingly, classes within *Firmicutes* (the other main monoderm phylum) showed two distinct responses: while *Bacilli* OTUs were overabundant under drought, *Clostridia* OTUs were depleted. Considering that the latter group is strictly anaerobic (28), an increase of oxygen levels after water withdrawal might have contributed to this differential response within the phylum. Additionally, several members of *Actinobacteria* are characterized by their filamentous growth habit and their ability to produce stress-resistant spores (29), two traits that could help this group resist drought stress. Other adaptive mechanisms to counteract water stress include osmoprotectant production, biofilm formation, and DNA repair upregulation (30), traits that could be prevalent in the set of microorganisms enriched under drought. Large-scale genome and metagenome sequencing of drought-enriched microbiota could further provide clues to possible functions that might contribute to tolerance to drought and other abiotic stresses in these taxa.

By simultaneously surveying the microbial communities assembled in the rhizosphere and endosphere, we were able to identify conserved and compartment-specific responses to drought. We found that a considerable fraction of OTUs affected by drought were only responsive in either the rhizosphere or the endosphere (Fig. 5A). Furthermore, OTUs that were exclusively drought-depleted in one compartment were also differentially abundant between the rhizosphere and endosphere of control plants, indicating that compartment-specific drought depletion is likely linked to preexisting compositional differences between compartments. In contrast, most OTUs that were drought enriched in only one compartment were not differentially abundant between the rhizospheres and endospheres of control plants. This last observation suggests that compartment-specific drought enrichment was not a product of predispositional differential abundances between compartments, but was rather a *de novo* restructuring of the rhizosphere and endosphere microbiota under drought pressure. The plant response to water deficit could have mediated some of these compartment-specific enrichments as drought triggers a complex molecular and physiological response to which associated microbes can actively react (31). For example, water stress can modify the amount and composition of root exudation (19, 20), which could potentially lead to the selective enrichment of certain microorganisms in the rhizosphere. Furthermore, roots can counteract the hyperosmotic conditions generated by drought through the synthesis and accumulation of osmolytes (32), a mechanism that allows plants to retain sufficient internal moisture to maintain their viability. Such plant responses could facilitate the enrichment of particular bacteria in the endosphere. Further studies (e.g., using mutants impaired in drought responses or drought-tolerant accessions) could shed light into these processes. We identified consistent genotypic differences in microbiota composition between the tested rice varieties, specifically between the *Oryza glaberrima* and *Oryza sativa* cultivars (Fig. 1D). Nevertheless, despite *O. glaberrima* being more drought tolerant than *O. sativa* (21), we were unable to identify a strong genotype-drought treatment interaction, and only a few individual OTUs were shown to have a differential response to drought based on genotype (Fig. S6). This suggests that the communities assembled in each cultivar responded consistently to the drought treatment. However, we cannot exclude subtle microbiome differences resulting as a product of genotype-treatment interaction that were not captured by this experimental design.

A central question is whether the observed drought-mediated changes in the root-associated communities are beneficial to the host plants, particularly in coping with drought stress. Plants grown in association with soil microbial communities that have previously experienced drought conditions have been shown to increase their host fitness under stress (33). Moreover, interactions with particular members of the microbiome can promote plant survival under water deficit conditions. Fungal symbionts have been shown to mediate stress tolerance through several mechanisms, including readjustment of the osmotic potential, increase in water use efficiency, and synthesis of antioxidant enzymes (34). Rice plants, in particular, have been reported to exhibit tolerance to salt and drought stress after being colonized by class 2 fungal endophytes (35). While we were not able to investigate the detailed taxonomic response to drought of fungal OTUs due to the large fraction of unclassified taxa, these limitations might be better addressed in future studies by expanding the databases used to assign taxonomies or by using alternative amplicons to ITS1 to survey the fungal diversity. In the case of bacteria, members of some of the drought-enriched taxa detected in our experiment have been identified as plant growth-promoting bacteria that can confer increased drought tolerance to the host. Multiple *Bacillus* species have been shown to promote drought resistance in various plant models, including *Arabidopsis* (36), *Brachypodium* (37), pepper (38), and rice (39). *Herbaspirillum seropedicae*, a species of the family *Oxalobacteraceae* (the main *Betaproteobacteria* drought-enriched family found in the endosphere of our samples) can help common beans recover from water stress (40). *Actinobacteria*, the phylum with the most prominent drought enrichment, is associated with the synthesis of antibiotics and other bioactive compounds, including growth-promoting substances (41, 42). Previous studies have implicated *Actinobacteria* in plant defense against fungi in wheat and strawberry, as well as promoting plant defense signaling in *Arabidopsis* (43, 44). Therefore, it is possible that the enrichment of *Actinobacteria* under drought confers advantages to the plant in defense against pathogens during a potentially debilitating environmental stress. In addition, the enrichment of specific bacterial taxa might also be a plant-directed mechanism to maintain an active microbiome in the face of an external challenge through promotion of specific taxa better adapted to surviving the stress. Definitive answers to these questions might require the development of functional assays, using systems in which microbial assemblages enriched by drought can be tested independently of the variables imposed by soil chemistry and structure.

## MATERIALS AND METHODS

### Experimental design.

All data presented in this article were generated from a greenhouse study carried out at University of California—Davis in the summer of 2016. In brief, four rice varieties grown in three different soils were exposed to two watering regimes. The experiment was set up following a split plot with factorial subplot design in which the watering regime was assigned to the main plots and combinations of soil type and cultivar were assigned to the subplots (Fig. S1A, B, and D). Specifically, eight 23-gallon plastic tubs were arranged in a 2-by-4 configuration, with each tub holding 15 5.5- by 5.5-inch pots: 12 to cover all combinations between the 4 cultivars and 3 soil types and 3 for unplanted bulk soils. Half of the tubs were subjected to a dry-down regime, while the rest remained under well-watered conditions. This design resulted in 4 biological replicates per soil-cultivar-treatment combination.

### Soil collection and processing.

The soils used in this experiment were collected from three geographically distant locations along the California Central Valley (Fig. S1C): an agricultural plot in Arbuckle (39°0′42.235″N, 121°55′19.632″W), a plot in the California Rice Experiment Station at Biggs (39°27′50.8″N, 121°44′14.4″W), and an experimental plot in Davis (38°32′37.91 N, 121°48′44.027″W). All soils were collected and transported to a greenhouse at University of California—Davis on 3 June 2016, where they remained stored until 20 June 2017. Individual soils were then thoroughly homogenized, scooped into 5.5- by 5.5-inch pots (40 pots per soil type, 2,000 g of soil per pot), and distributed across 8 23-gallon plastic tubs. Prior to seedling transplantation, enough water was added to the tubs to submerge the soils.

### Seed germination and plant growth.

The varieties used in this experiment spanned the two species of domesticated rice: *Oryza sativa* was represented by the accessions M206 (subsp. *japonica*) and IR20 (subsp. *indica*), while *Oryza glaberrima* was represented by the accessions TOg7102 and CG14. For each variety, dehulled seeds were surface sterilized (50% bleach for 5 min, followed by washes with autoclaved water), plated on Murashige and Skoog (MS) agar, and germinated in a growth chamber for 7 days. On 22 June 2016, axenic seedlings were transplanted to pots in the greenhouse, where they were irrigated with deionized water every other day to keep the soil under submergence. Nutrient water was supplied on two occasions: 7 and 21 days after transplantation.

### Drought treatment.

Four weeks after the seedlings were transplanted, water was drained from the plastic tubs, and soils were allowed to dry until half of the plants exhibited drought stress symptoms (leaf curling and senescence), which occurred 5 days after water withdrawal. At this point, 1 liter of deionized water was added to the tubs every other day, which kept the plants alive (as evidenced by plants recovering their turgidity a few hours after water addition) but under stress (as evidenced by the resurgence of wilting 2 days after water addition). For control plants, tubs were irrigated every other day to keep the soil under submergence. Three weeks after water withdrawal, samples were harvested.

### Sample collection, processing, and DNA extraction.

Samples were collected over a 2-day period (10 to 11 August 2016). Each day, four full tubs were processed: two for each watering regimen to guarantee that all factor combinations were equally represented. Sample collection and compartment separation were done following a previously described protocol (5). Briefly, harvested roots were vigorously shaken to remove loose soil and then placed into 50-ml Falcon tubes with 15 ml of autoclaved phosphate-buffered saline (PBS) solution. For consistency, we only collected the 5 cm of root immediately below the root-shot junction (Fig. S1E). The rhizospheric compartment was separated by thoroughly vortexing the roots and collecting 500 µl of the resulting soil suspension in PowerBead tubes (Mo Bio Laboratories). For collection of the endosphere compartment, roots were thoroughly washed in fresh PBS to further discard any remaining soil and sonicated three times (50 to 60 Hz for 30 s) to remove the rhizoplane microorganisms. Sonicated roots were placed in PowerBead tubes and homogenized by intense agitation for 1 min (Mini Beadbeater; BioSpec Products). Bulk soil samples were collected ~5 cm below the soil surface. DNA extractions were performed immediately after compartment separation, following the PowerSoil DNA isolation kit (Mo Bio Laboratories) protocol.

### Water levels.

For each pot, soil samples were collected at the end of experiment on 50-ml Falcon tubes. After recording the initial weight, samples were placed and left inside a 42°C oven for 4 months. The dry weight of the samples was then recorded, and the percentage of moisture was calculated.

### Library construction and sequencing.

Library construction followed a previously described dual-indexing strategy (5, 45). For 16S rRNA gene libraries, the V4 region was amplified using the universal primers 515F and 806R. Amplification was carried out with the following touchdown PCR program: a first phase consisting of 95°C for 5 min, followed by 7 cycles of 95°C for 45 s, 65°C for 1 min (decreasing at 2°C/cycle), and 72°C for 90 s, with a second phase consisting of 30 cycles of 95°C for 45 s, 50°C for 30 s, and 72°C for 90 s, followed by a final extension at 72°C for 10 min. For fungal libraries, the ITS1 region was amplified using the universal primers ITS1-F and ITS2 (24, 25, 46). Amplification was carried out with the following touchdown PCR program: a first phase consisting of 95°C for 5 min, followed by 35 cycles of 95°C for 45 s, 50°C for 60 s, and 72°C for 60 s, followed by a final extension at 72°C for 10 min. All PCR amplifications were performed using the HotStar HiFidelity polymerase kit (Qiagen) with the following components: 6.25 µl H_2_O, 2.5-µl HotStar PCR buffer, 1.25 µl forward primer (10 µM), 1.25 µl reverse primer (10 µM), 1 µl template DNA, and 0.25 µl HotStar polymerase. After running a 1% agarose gel to verify proper amplification, libraries were cleaned with AmPure XP magnetic beads (Beckman Coulter, Inc.), quantified (Qubit dsDNA HS assay kit; Thermo Fisher Scientific), and pooled in equimolar concentrations. Pooled libraries were then concentrated, gel purified (Nucleoscopic gel and PCR cleanup kit; Macherey-Nagel), quality checked (BioAnalyzer HS DNA kit; Agilent Technologies), and submitted for 2- by 250-bp Miseq sequencing (Illumina) to the DNA Technologies and Expression Analysis Cores at the UC Davis Genome Center (supported by NIH Shared Instrumentation Grant 1S10OD010786-01).

### Sequence processing for 16S.

The paired-end reads were demultiplexed with custom scripts (https://github.com/RiceMicrobiome/Edwards-et-al.-2014/tree/master/sequencing_scripts) and assembled into single sequences with PANDAseq (47). Chimeric sequences were detected and discarded with usearch61 (48). OTU clustering at 97% identity was performed with the QIIME (45) implementation of UCLUST (48), using an open reference strategy against the 13_8 release of the Greengenes 16S sequence database (49). Representative sequences were then aligned with PyNAST (50), and a phylogenetic tree was built with FastTree (51). OTUs classified as mitochondria and chloroplast were discarded from the OTU table, and nonprevalent OTUs (defined as OTUs not present in at least 5% of our samples) were filtered out.

### Sequence processing for ITS1.

The paired-end reads were demultiplexed with the custom scripts mentioned above, and primer regions were removed with Cutadapt (52), with a required minimum overlap of 10, a minimum final sequence length of 10, and a quality trim score of 10. Reads were then assembled into single sequences with PANDAseq (47), and the flanking ribosomal small subunit and 5.8S regions were removed with ITSx (53). Chimeric sequences were detected and discarded with usearch61 (48). OTU clustering at 97% identity was performed with the QIIME (45) implementation of UCLUST (48), using an open reference strategy against version 7 of the UNITE database (54) and allowing reverse strand matching. Taxonomy assignment was performed at a 0.65 similarity threshold. OTUs classified as *Plantae* or *Protista* were discarded from the OTU table, and nonprevalent OTUs (defined as OTUs not present in at least 5% of our samples) were filtered from the data set before analysis. The consensus sequences of the five most abundant OTUs without an assigned classification at the kingdom level were aligned against the NCBI nr/nt database using the BLAST algorithm. The top three and the fifth unclassified OTUs returned best hits to ribosomal RNAs of uncultured fungi (E values ranging from 4e−23 to 2e−50), while the fourth unclassified OTU returned no significant hits.

### Statistical analyses.

All analyses were conducted in the R Environment version 3.2.3 (55). For beta-diversity analyses (i.e., PERMANOVA, CAP, and PCoA), OTU counts were normalized using the variance-stabilizing transformation implemented in DESeq2 (56, 57). Weighted UniFrac distances (58) were then calculated with phyloseq (59). Unconstrained principal-coordinate analysis was performed with the pcoa function from the ape package (60). The adonis and capscale functions from the vegan package (61) were used to perform permutational multivariate analyses of variance and canonical analyses of principal coordinates, respectively. For phylum-level analyses, raw OTU counts were collapsed by phylum or, in the case of *Proteobacteria*, by class. Differential abundance analyses were performed with DESeq2 (56, 57) for both OTUs and phyla. All plots were generated with the ggplot2 package (62), except for the taxonomic dendrograms, which were plotted with graphlan (63), and the Venn diagrams, which were plotted with the VennDiagram package. All scripts and intermediate files have been deposited in GitHub (https://github.com/cmsantosm/Drought-Root-Microbiome).

### Accession number(s).

Raw reads have been deposited in the Short Read Archive of NCBI under project no. PRJNA386367.

## References

[1] Friesen ML, Porter SS, Stark SC, Von Wettberg EJ, Sachs JL, Martinez-Romero E 2011 Microbially mediated plant functional traits. Annu Rev Ecol Evol Syst 42:23–46. doi:10.1146/annurev-ecolsys-102710-145039.

[2] Lundberg DS, Lebeis SL, Paredes SH, Yourstone S, Gehring J, Malfatti S, Tremblay J, Engelbrektson A, Kunin V, del Rio TG, Edgar RC, Eickhorst T, Ley RE, Hugenholtz P, Tringe SG, Dangl JL 2012 Defining the core Arabidopsis thaliana root microbiome. Nature 488:86–90. doi:10.1038/nature11237. 22859206 PMC4074413

[3] Bulgarelli D, Rott M, Schlaeppi K, Ver Loren van Themaat E, Ahmadinejad N, Assenza F, Rauf P, Huettel B, Reinhardt R, Schmelzer E, Peplies J, Gloeckner FO, Amann R, Eickhorst T, Schulze-Lefert P 2012 Revealing structure and assembly cues for Arabidopsis root-inhabiting bacterial microbiota. Nature 488:91–95. doi:10.1038/nature11336. 22859207

[4] Peiffer JA, Spor A, Koren O, Jin Z, Tringe SG, Dangl JL, Buckler ES, Ley RE 2013 Diversity and heritability of the maize rhizosphere microbiome under field conditions. Proc Natl Acad Sci U S A 110:6548–6553. doi:10.1073/pnas.1302837110. 23576752 PMC3631645

[5] Edwards J, Johnson C, Santos-Medellín C, Lurie E, Podishetty NK, Bhatnagar S, Eisen JA, Sundaresan V 2015 Structure, variation, and assembly of the root-associated microbiomes of rice. Proc Natl Acad Sci U S A 112:E911–E920. doi:10.1073/pnas.1414592112. 25605935 PMC4345613

[6] Coleman-Derr D, Desgarennes D, Fonseca-Garcia C, Gross S, Clingenpeel S, Woyke T, North G, Visel A, Partida-Martinez LP, Tringe SG 2016 Plant compartment and biogeography affect microbiome composition in cultivated and native *Agave* species. New Phytol 209:798–811. doi:10.1111/nph.13697. 26467257 PMC5057366

[7] Wagner MR, Lundberg DS, del Rio TG, Tringe SG, Dangl JL, Mitchell-Olds T 2016 Host genotype and age shape the leaf and root microbiomes of a wild perennial plant. Nat Commun 7:12151. doi:10.1038/ncomms12151. 27402057 PMC4945892

[8] Van Der Heijden MGA, Schlaeppi K 2015 Root surface as a frontier for plant microbiome research. Proc Natl Acad Sci U S A 112:2299–2300. doi:10.1073/pnas.1500709112. 25713090 PMC4345591

[9] Busby PE, Soman C, Wagner MR, Friesen ML, Kremer J, Bennett A, Morsy M, Eisen JA, Leach JE, Dangl JL 2017 Research priorities for harnessing plant microbiomes in sustainable agriculture. PLoS Biol 15:e2001793. doi:10.1371/journal.pbio.2001793. 28350798 PMC5370116

[10] Rodriguez RJ, Henson J, Van Volkenburgh E, Hoy M, Wright L, Beckwith F, Kim YO, Redman RS 2008 Stress tolerance in plants via habitat-adapted symbiosis. ISME J 2:404–416. doi:10.1038/ismej.2007.106. 18256707

[11] Lesk C, Rowhani P, Ramankutty N 2016 Influence of extreme weather disasters on global crop production. Nature 529:84–87. doi:10.1038/nature16467. 26738594

[12] Core Writing Team, Pachauri RK, Meyer LA (ed) 2014 Climate change 2014: synthesis report. Contribution of working groups I, II and III to the fifth assessment report of the Intergovernmental Panel on Climate Change. IPCC, Geneva, Switzerland.

[13] Barnard RL, Osborne CA, Firestone MK 2013 Responses of soil bacterial and fungal communities to extreme desiccation and rewetting. ISME J 7:2229–2241. doi:10.1038/ismej.2013.104. 23823489 PMC3806258

[14] Bouskill NJ, Lim HC, Borglin S, Salve R, Wood TE, Silver WL, Brodie EL 2013 Pre-exposure to drought increases the resistance of tropical forest soil bacterial communities to extended drought. ISME J 7:384–394. doi:10.1038/ismej.2012.113. 23151641 PMC3554394

[15] Xu Z, Zhou G, Shimizu H 2010 Plant responses to drought and rewatering. Plant Signal Behav 5:649–654. doi:10.4161/psb.5.6.11398. 20404516 PMC3001553

[16] Gray SB, Brady SM 2016 Plant developmental responses to climate change. Dev Biol 419:64–77. doi:10.1016/j.ydbio.2016.07.023. 27521050

[17] Smith S, De Smet I 2012 Root system architecture: insights from Arabidopsis and cereal crops. Philos Trans R Soc Lond B Biol Sci 367:1441–1452. doi:10.1098/rstb.2011.0234. 22527386 PMC3321685

[18] Hasibeder R, Fuchslueger L, Richter A, Bahn M 2015 Summer drought alters carbon allocation to roots and root respiration in mountain grassland. New Phytol 205:1117–1127. doi:10.1111/nph.13146. 25385284 PMC4303983

[19] Song F, Han X, Zhu X, Herbert SJ 2012 Response to water stress of soil enzymes and root exudates from drought and non-drought tolerant corn hybrids at different growth stages. Can J Soil Sci 92:501–507. doi:10.4141/cjss2010-057.

[20] Henry A, Doucette W, Norton J, Bugbee B 2007 Changes in crested wheatgrass root exudation caused by flood, drought, and nutrient stress. J Environ Qual 36:904–912. doi:10.2134/jeq2006.0425sc. 17485723

[21] Ndjiondjop MN, Manneh B, Cissoko M, Drame NK, Kakai RG, Bocco R, Baimey H, Wopereis M 2010 Drought resistance in an interspecific backcross population of rice (Oryza spp.) derived from the cross WAB56-104 (O. sativa)×CG14 (O. glaberrima). Plant Sci 179:364–373. doi:10.1016/j.plantsci.2010.06.006.

[22] O’Toole JC, Cruz RT, Singh TN 1979 Leaf rolling and transpiration. Plant Sci Lett 16:111–114. doi:10.1016/0304-4211(79)90015-4.

[23] Philippot L, Andersson SGE, Battin TJ, Prosser JI, Schimel JP, Whitman WB, Hallin S 2010 The ecological coherence of high bacterial taxonomic ranks. Nat Rev Microbiol 8:523–529. doi:10.1038/nrmicro2367. 20531276

[24] Gardes M, Bruns TD 1993 ITS primers with enhanced specificity for basidiomycetes—application to the identification of mycorrhizae and rusts. Mol Ecol 2:113–118. doi:10.1111/j.1365-294X.1993.tb00005.x. 8180733

[25] White TJ, Bruns T, Lee S, Taylor J 1990 Amplification and direct sequencing of fungal ribosomal RNA genes for phylogenetics, p 315–322. *In* Innis MA, Gelfand DH, Sninsky JJ, White TJ (ed), PCR protocols: a guide to methods and applications. Academic Press, New York, NY.

[26] Amend AS, Martiny AC, Allison SD, Berlemont R, Goulden ML, Lu Y, Treseder KK, Weihe C, Martiny JB 2016 Microbial response to simulated global change is phylogenetically conserved and linked with functional potential. ISME J 10:109–118. doi:10.1038/ismej.2015.96. 26046258 PMC4681869

[27] Lennon JT, Aanderud ZT, Lehmkuhl BK, Schoolmaster DR 2012 Mapping the niche space of soil microorganisms using taxonomy and traits. Ecology 93:1867–1879. doi:10.1890/11-1745.1. 22928415

[28] Caumette P, Brochier-Armanet C, Normand P 2015 Taxonomy and phylogeny of prokaryotes 6, p 145–190. *In* Bertrand J-C, Caumette P, Lebaron P, Matheron R, Normand P, Simen-Ngando T (ed), Environmental microbiology: fundamentals and applications. Springer, Dordrecht, Netherlands.

[29] Barka EA, Vatsa P, Sanchez L, Gaveau-Vaillant N, Jacquard C, Klenk HP, Clément C, Ouhdouch Y, van Wezel GP 2016 Taxonomy, physiology, and natural products of Actinobacteria. Microbiol Mol Biol Rev 80:1–43. doi:10.1128/MMBR.00019-15. 26609051 PMC4711186

[30] Lebre PH, De Maayer P, Cowan DA 2017 Xerotolerant bacteria: surviving through a dry spell. Nat Rev Microbiol 15:285–296. doi:10.1038/nrmicro.2017.16. 28316329

[31] Sheibani-Tezerji R, Rattei T, Sessitsch A, Trognitz F, Mitter B 2015 Transcriptome profiling of the endophyte Burkholderia phytofirmans PsJN indicates sensing of the plant environment and drought stress. mBio 6:e00621-15. doi:10.1128/mBio.00621-15. 26350963 PMC4600099

[32] Janiak A, Kwaśniewski M, Szarejko I 2016 Gene expression regulation in roots under drought. J Exp Bot 67:1003–1014. doi:10.1093/jxb/erv512. 26663562

[33] Lau JA, Lennon JT 2012 Rapid responses of soil microorganisms improve plant fitness in novel environments. Proc Natl Acad Sci U S A 109:14058–14062. doi:10.1073/pnas.1202319109. 22891306 PMC3435152

[34] Singh LP, Gill SS, Tuteja N 2011 Unraveling the role of fungal symbionts in plant abiotic stress tolerance. Plant Signal Behav 6:175–191. doi:10.4161/psb.6.2.14146. 21512319 PMC3121976

[35] Redman RS, Kim YO, Woodward CJDA, Greer C, Espino L, Doty SL, Rodriguez RJ 2011 Increased fitness of rice plants to abiotic stress via habitat adapted symbiosis: a strategy for mitigating impacts of climate change. PLoS One 6:e14823. doi:10.1371/journal.pone.0014823. 21750695 PMC3130040

[36] Zhou C, Ma Z, Zhu L, Xiao X, Xie Y, Zhu J, Wang J 2016 Rhizobacterial strain Bacillus megaterium BOFC15 induces cellular polyamine changes that improve plant growth and drought resistance. Int J Mol Sci 17:976. doi:10.3390/ijms17060976. PMC4926508 27338359

[37] Gagné-Bourque F, Mayer BF, Charron JB, Vali H, Bertrand A, Jabaji S 2015 Accelerated growth rate and increased drought stress resilience of the model grass Brachypodium distachyon colonized by *Bacillus subtilis* B26. PLoS One 10:e0130456. doi:10.1371/journal.pone.0130456. 26103151 PMC4477885

[38] Marasco R, Rolli E, Ettoumi B, Vigani G, Mapelli F, Borin S, Abou-Hadid AF, El-Behairy UA, Sorlini C, Cherif A, Zocchi G, Daffonchio D 2012 A drought resistance-promoting microbiome is selected by root system under desert farming. PLoS One 7:e48479. doi:10.1371/journal.pone.0048479. 23119032 PMC3485337

[39] Kakar KU, Ren XL., Nawaz Z, Cui Z-Q, Li B, Xie G-L, Hassan MA, Ali E, Sun G-C 2016 A consortium of rhizobacterial strains and biochemical growth elicitors improve cold and drought stress tolerance in rice (*Oryza sativa* L.). Plant Biol 18:471–483. doi:10.1111/plb.12427. 26681628

[40] da Piedade Melo A, Olivares FL, Médici LO, Torres-Neto A, Dobbss LB, Canellas LP 2017 Mixed rhizobia and Herbaspirillum seropedicae inoculations with humic acid-like substances improve water-stress recovery in common beans. Chem Biol Technol Agric 4:6. doi:10.1186/s40538-017-0090-z.

[41] Rey T, Dumas B 2017 Plenty is no plague: Streptomyces symbiosis with crops. Trends Plant Sci 22:30–37. doi:10.1016/j.tplants.2016.10.008. 27916552

[42] Palaniyandi SA, Yang SH, Zhang L, Suh JW 2013 Effects of Actinobacteria on plant disease suppression and growth promotion. Appl Microbiol Biotechnol 97:9621–9636. doi:10.1007/s00253-013-5206-1. 24092003

[43] Conn VM, Walker AR, Franco CMM 2008 Endophytic actinobacteria induce defense pathways in *Arabidopsis thaliana*. Mol Plant Microbe Interact 21:208–218. doi:10.1094/MPMI-21-2-0208. 18184065

[44] Cha JY, Han S, Hong HJ, Cho H, Kim D, Kwon Y, Kwon SK, Crüsemann M, Bok Lee Y, Kim JF, Giaever G, Nislow C, Moore BS, Thomashow LS, Weller DM, Kwak YS 2016 Microbial and biochemical basis of a Fusarium wilt-suppressive soil. ISME J 10:119–129. doi:10.1038/ismej.2015.95. 26057845 PMC4681868

[45] Caporaso JG, Kuczynski J, Stombaugh J, Bittinger K, Bushman FD, Costello EK, Fierer N, Peña AG, Goodrich JK, Gordon JI, Huttley GA, Kelley ST, Knights D, Koenig JE, Ley RE, Lozupone CA, McDonald D, Muegge BD, Pirrung M, Reeder J, Sevinsky JR, Turnbaugh PJ, Walters WA, Widmann J, Yatsunenko T, Zaneveld J, Knight R 2010 QIIME allows analysis of high-throughput community sequencing data. Nat Methods 7:335–336. doi:10.1038/nmeth.f.303. 20383131 PMC3156573

[46] Agler MT, Ruhe J, Kroll S, Morhenn C, Kim ST, Weigel D, Kemen EM 2016 Microbial hub taxa link host and abiotic factors to plant microbiome variation. PLoS Biol 14:e1002352. doi:10.1371/journal.pbio.1002352. 26788878 PMC4720289

[47] Masella AP, Bartram AK, Truszkowski JM, Brown DG, Neufeld JD 2012 PANDAseq: paired-end assembler for Illumina sequences. BMC Bioinformatics 13:31. doi:10.1186/1471-2105-13-31. 22333067 PMC3471323

[48] Edgar RC 2010 Search and clustering orders of magnitude faster than BLAST. Bioinformatics 26:2460–2461. doi:10.1093/bioinformatics/btq461. 20709691

[49] DeSantis TZ, Hugenholtz P, Larsen N, Rojas M, Brodie EL, Keller K, Huber T, Dalevi D, Hu P, Andersen GL 2006 Greengenes, a chimera-checked 16S rRNA gene database and workbench compatible with ARB. Appl Environ Microbiol 72:5069–5072. doi:10.1128/AEM.03006-05. 16820507 PMC1489311

[50] Caporaso JG, Bittinger K, Bushman FD, Desantis TZ, Andersen GL, Knight R 2010 PyNAST: a flexible tool for aligning sequences to a template alignment. Bioinformatics 26:266–267. doi:10.1093/bioinformatics/btp636. 19914921 PMC2804299

[51] Price MN, Dehal PS, Arkin AP 2009 Fasttree: computing large minimum evolution trees with profiles instead of a distance matrix. Mol Biol Evol 26:1641–1650. doi:10.1093/molbev/msp077. 19377059 PMC2693737

[52] Martin M 2011 Cutadapt removes adapter sequences from high-throughput sequencing reads. EMBnet J 17:10. doi:10.14806/ej.17.1.200.

[53] Bengtsson-Palme J, Ryberg M, Hartmann M, Branco S, Wang Z, Godhe A, De Wit P, Sánchez-García M, Ebersberger I, de Sousa F, Amend AS, Jumpponen A, Unterseher M, Kristiansson E, Abarenkov K, Bertrand YJK, Sanli K, Eriksson KM, Vik U, Veldre V, Nilsson RH 2013 Improved software detection and extraction of ITS1 and ITS2 from ribosomal ITS sequences of fungi and other eukaryotes for analysis of environmental sequencing data. Methods Ecol Evol 4:914–919. doi:10.1111/2041-210X.12073.

[54] Kõljalg U, Nilsson RH, Abarenkov K, Tedersoo L, Taylor AFS, Bahram M, Bates ST, Bruns TD, Bengtsson-Palme J, Callaghan TM, Douglas B, Drenkhan T, Eberhardt U, Dueñas M, Grebenc T, Griffith GW, Hartmann M, Kirk PM, Kohout P, Larsson E, Lindahl BD, Lücking R, Martín MP, Matheny PB, Nguyen NH, Niskanen T, Oja J, Peay KG, Peintner U, Peterson M, Põldmaa K, Saag L, Saar I, Schüßler A, Scott JA, Senés C, Smith ME, Suija A, Taylor DL, Telleria MT, Weiss M, Larsson KH 2013 Towards a unified paradigm for sequence-based identification of fungi. Mol Ecol 22:5271–5277. doi:10.1111/mec.12481. 24112409

[55] R Core Team 2015 R: a language and environment for statistical computing. R Foundation for Statistical Computing, Vienna, Austria https://www.r-project.org/.

[56] Love MI, Huber W, Anders S 2014 Moderated estimation of fold change and dispersion for RNA-seq data with DESeq2. Genome Biol 15:550. doi:10.1186/s13059-014-0550-8. 25516281 PMC4302049

[57] McMurdie PJ, Holmes S 2014 Waste not, want not: why rarefying microbiome data is inadmissible. PLoS Comput Biol 10:e1003531. doi:10.1371/journal.pcbi.1003531. 24699258 PMC3974642

[58] Lozupone C, Knight R 2005 UniFrac: a new phylogenetic method for comparing microbial communities. Appl Environ Microbiol 71:8228–8235. doi:10.1128/AEM.71.12.8228-8235.2005. 16332807 PMC1317376

[59] McMurdie PJ, Holmes S 2013 phyloseq: an R package for reproducible interactive analysis and graphics of microbiome census data. PLoS One 8:e61217. doi:10.1371/journal.pone.0061217. 23630581 PMC3632530

[60] Paradis E, Claude J, Strimmer K 2004 APE: analyses of phylogenetics and evolution in R language. Bioinformatics 20:289–290. doi:10.1093/bioinformatics/btg412. 14734327

[61] Oksanen J, Blanchet FG, Friendly M, Kindt R, Legendre P, Mcglinn D, Minchin PR, O’hara RB, Simpson GL, Solymos P, Henry M, Stevens H, Szoecs E, Wagner H 2017 vegan: Community Ecology Package, 2.4-2. https://cran.r-project.org.

[62] Wickham H 2009 ggplot2: elegant graphics for data analysis. Springer-Verlag, New York, NY.

[63] Asnicar F, Weingart G, Tickle TL, Huttenhower C, Segata N 2015 Compact graphical representation of phylogenetic data and metadata with GraPhlAn. PeerJ 3:e1029. doi:10.7717/peerj.1029. 26157614 PMC4476132

