# Supplementary material for: Drought Stress Results in a Compartment-Specific Restructuring of the Rice Root-Associated Microbiomes
Source: mBio. 2017 Jul 18;8(4):e00764-17. doi: 10.1128/mBio.00764-17 (PMC5516253; doi:10.1128/mBio.00764-17)
Supplement: FIG S2 [file mbo004173388sf2.pdf]

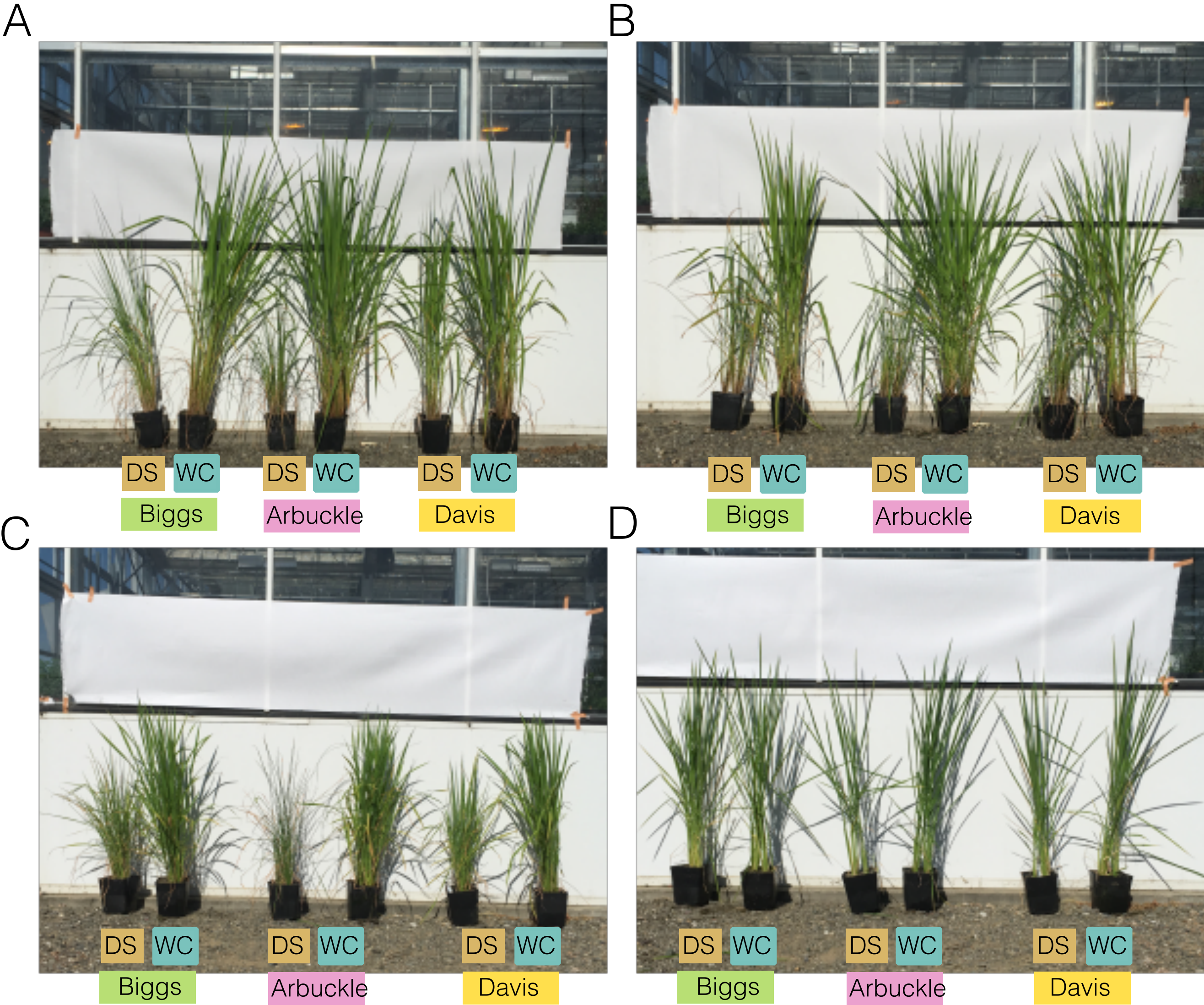
A
B
C
D
DS
WC
Biggs
Arbuckle
Davis
