# Supplementary material for: Drought Stress Results in a Compartment-Specific Restructuring of the Rice Root-Associated Microbiomes
Source: mBio. 2017 Jul 18;8(4):e00764-17. doi: 10.1128/mBio.00764-17 (PMC5516253; doi:10.1128/mBio.00764-17)
Supplement: TABLE S1 [file mbo004173388st1.pdf]

Table 1

A) Soil water content of drought and control samples at the end of the experiment

Soil type	Cultivar	Soil water content under well-watered conditions (%)	Soil water content under drought conditions (%)	$\frac{(\text{Control - Drought})}{\text{Control}}$
Arbuckle	Bulk Soil	36.65 +/- 1.28	33.03 +/- 1	9.87
Arbuckle	G1	33.77 +/- 1.09	10.99 +/- 0.78	67.46
Arbuckle	G2	33.92 +/- 0.99	10.96 +/- 1.2	67.68
Arbuckle	S1	33.32 +/- 0.52	11.99 +/- 1.59	64.00
Arbuckle	S2	33.86 +/- 0.28	15.79 +/- 5.87	53.36
Biggs	Bulk Soil	33.39 +/- 2.52	31.35 +/- 1.16	6.13
Biggs	G1	32.78 +/- 1.03	9.74 +/- 0.44	70.30
Biggs	G2	32.46 +/- 1.45	9.43 +/- 1.26	70.94
Biggs	S1	32.27 +/- 1.33	17.02 +/- 8.09	47.25
Biggs	S2	31.58 +/- 1.53	21.61 +/- 5.54	31.59
Davis	Bulk Soil	29.64 +/- 1.36	26.27 +/- 1.27	11.37
Davis	G1	28.42 +/- 1.37	8.69 +/- 2.01	69.43
Davis	G2	29.88 +/- 1.61	8.45 +/- 0.78	71.73
Davis	S1	29.96 +/- 1.84	9.81 +/- 0.87	67.24
Davis	S2	30.53 +/- 1.51	16.25 +/- 5.95	46.77

B) ANOVA testing the effect of watering treatment, soil type, and cultivar on the water content of soils at the end of the experiment. Bulk soil samples were excluded before running the analysis because there is no cultivar assigned to them.

Df	SumsOfSqs	MeanSqs	F.Model	R2	Pr(>F)
Treatment	1	8971.399	8971.399	1089.597	0.000
Soil	2	189.636	94.818	11.516	0.000

Cultivar	3	275.928	91.976	11.171	0.000
Treatment:Soil	2	50.179	25.089	3.047	0.054
Treatment:Cultivar	3	262.189	87.396	10.614	0.000
Soil:Cultivar	6	48.223	8.037	0.976	0.448
Treatment:Soil:Cultivar	6	73.622	12.270	1.490	0.194
Residuals	72	592.825	8.234		
