# Supplementary material for: Drought Stress Results in a Compartment-Specific Restructuring of the Rice Root-Associated Microbiomes
Source: mBio. 2017 Jul 18;8(4):e00764-17. doi: 10.1128/mBio.00764-17 (PMC5516253; doi:10.1128/mBio.00764-17)
Supplement: TABLE S2 [file mbo004173388st2.pdf]

Table 2

A) PERMANOVA testing the effect of soil, compartment, and watering treatment on beta-diversity.

term	df	SumsOfSqs	MeanSqs	F.Model	R2	p.value
Compartment	2	2.394	1.197	93.361	0.285	**0.001**
Soil	2	2.323	1.161	90.597	0.277	**0.001**
Treatment	1	0.830	0.830	64.715	0.099	**0.001**
Library	1	0.017	0.017	1.362	0.002	0.196
Compartment:Treatment	2	0.165	0.082	6.429	0.020	**0.001**
Soil:Treatment	2	0.032	0.016	1.229	0.004	0.228
Residuals	205	2.628	0.013	NA	0.313	NA
Total	215	8.388	NA	NA	1.000	NA

B) Simple effects testing the effect of drought within each level of compartment.

term	df	SumsOfSqs	MeanSqs	F.Model	R2	p.value
Compartment	2	2.394	1.197	93.027	0.285	**0.001**
Soil	2	2.323	1.161	90.272	0.277	**0.001**
trt.in.bs	1	0.064	0.064	4.985	0.008	**0.001**
trt.in.rs	1	0.434	0.434	33.716	0.052	**0.001**
trt.in.es	1	0.497	0.497	38.666	0.059	**0.001**
Residuals	208	2.676	0.013	NA	0.319	NA
Total	215	8.388	NA	NA	1.000	NA

C) PERMANOVA testing the effect of soil, compartment, watering treatment, and cultivar on beta-diversity. Bulk soils were excluded before running the analysis because they don’t have a cultivar assigned.

term	df	SumsOfSqs	MeanSqs	F.Model	R2	p.value
Compartment	1	1.872	1.872	151.321	0.252	**0.001**
Soil	2	2.146	1.073	86.744	0.289	**0.001**
Treatment	1	0.833	0.833	67.293	0.112	**0.001**

Cultivar	3	0.173	0.058	4.669	0.023	**0.001**
Library	1	0.015	0.015	1.190	0.002	0.263
Compartment:Treatment	1	0.099	0.099	7.984	0.013	**0.001**
Soil:Treatment	2	0.036	0.018	1.470	0.005	0.145
Treatment:Cultivar	3	0.063	0.021	1.690	0.008	**0.039**
Residuals	177	2.190	0.012	NA	0.295	NA
Total	191	7.426	NA	NA	1.000	NA
